# An Effective Strategy to Maintain the CALPHAD Atomic Mobility Database of Multicomponent Systems and Its Application to Hcp Mg–Al–Zn–Sn Alloys

**DOI:** 10.3390/ma15010283

**Published:** 2021-12-31

**Authors:** Ting Cheng, Jing Zhong, Lijun Zhang

**Affiliations:** State Key Laboratory of Powder Metallurgy, Central South University, Changsha 410083, China; chengting@csu.edu.cn (T.C.); zhongjingjogy@csu.edu.cn (J.Z.)

**Keywords:** atomic mobility, CALPHAD, diffusion couple, HitDIC, Hcp Mg–Al–Zn–Sn alloys

## Abstract

In this paper, a general and effective strategy was first developed to maintain the CALPHAD atomic mobility database of multicomponent systems, based on the pragmatic numerical method and freely accessible HitDIC software, and then applied to update the atomic mobility descriptions of the hcp Mg–Al–Zn, Mg–Al–Sn, and Mg–Al–Zn–Sn systems. A set of the self-consistent atomic mobility database of the hcp Mg–Al–Zn–Sn system was established following the new strategy presented. A comprehensive comparison between the model-predicted composition–distance profiles/inter-diffusivities in the hcp Mg–Al–Zn, Mg–Al–Sn, and Mg–Al–Zn–Sn systems from the presently updated atomic mobilities and those from the previous ones that used the traditional method indicated that significant improvement can be achieved utilizing the new strategy, especially in the cases with sufficient experimental composition–distance profiles and/or in higher-order systems. Furthermore, it is anticipated that the proposed strategy can serve as a standard for maintaining the CALPHAD atomic mobility database in different multicomponent systems.

## 1. Introduction

As is well known, the mechanical properties of metallic materials, such as strength, ductility, and hardness, are closely related to their microstructural formation during various preparation processes such as solidification, solid solution, and aging [1,2]. To achieve a comprehensive understanding of different preparation processes, accurate diffusion coefficients of composition and temperature dependence should be the prerequisite. For typical multicomponent technical alloys, direct experimental measurement of the complex diffusion coefficient matrices seems to be very difficult [3]. One alternative substitution in the CALPHAD (CALculation of PHAse Diagram) community is to predict a variety of composition- and temperature-dependent diffusion coefficients from the established atomic mobility database of the target alloys together with the corresponding thermodynamic database [4].

In terms of the CALPHAD framework, the traditional procedure for establishing the atomic mobility database of multicomponent alloys is referred to Figure 6.3 of a recent book chapter by Zhang and Chen [5], and it is also briefly described as follows: (*i*) Step 1: conduct a literature review of various diffusion properties in boundary unary, binary, ternary, and higher-order systems including the diffusion coefficients such as self/impurity diffusion coefficients for unary systems, interdiffusion coefficients for binary and ternary systems, and tracer coefficients for binary and higher-order systems as well as the experimental composition–distance profiles for quaternary and higher-order systems; (*ii*) Step 2: supplement the diffusion coefficients in boundary unary, binary, and ternary systems lacking the diffusion coefficients. The self/impurity diffusion coefficients of unary systems and tracer diffusion coefficients for binary and higher-order systems can be determined by the tracer method [6], first-principles [7], molecular dynamics [8], and also some indirect methods [9,10]. The interdiffusion coefficients in binary and ternary systems can be determined by either traditional Matano methods [11,12,13,14,15] or numerical inverse methods [16,17,18,19]. (*iii*) Step 3: Select the reasonable diffusion model(s) for the target phase(s). For detail information on this aspect, please also refer to the recent book chapter [5]. (*iv*) Step 4: Assess the atomic mobilities from the unary to binary and then ternary systems. (*v*) Extrapolate and validate the atomic mobilities of quaternary and higher-order systems, which can be directly extrapolated from those of boundary ternary systems, and then validated by comparing the model-predicted composition–distance profiles with the experimental ones. If most of the predicted results are inconsistent with the experimental data, Step 4 (maybe together with Step 3) should be repeated, until good agreement between the predicted and experimental observations of higher-order systems is achieved.

After the first version of the CALPHAD atomic mobility database is established, maintenance of the released database is essential, because some new experimental observations and theoretical calculations are likely to be produced from time to time. In general, for a technologically important multicomponent system, the atomic mobility descriptions in boundary unary and binary systems are typically reasonable, since sufficient and reliable diffusion coefficients are usually available [20]; thus, there is no need to update those atomic mobility descriptions frequently. For the boundary ternary systems, only scattered experimental interdiffusion coefficients are available in most cases due to the low efficiency of the Matano–Kirkaldy (M–K) method [12] with which only four independent interdiffusion coefficients can be obtained at the intersection point from the diffusion paths of two diffusion couples [21]. In order to improve the quality of the atomic mobility database, more interdiffusion coefficients covering wider compositions and temperature ranges are indispensable. Thus, continuous renewal of the corresponding atomic mobilities is necessary, but it is a really time- and cost-consuming process. While for the boundary quaternary and higher-order systems, some new experimental composition–distance profiles from the diffusion couples/multiples may appear and only be used to validate the established atomic mobility database, it cannot be directly employed to update the database [21] according to the traditional approach to CALPHAD database development. Therefore, there is an urgent need to improve the current situation.

One more superior approach is to utilize the numerical inverse method for maintaining the atomic mobility database of the target multicomponent system. Very recently, two of the present authors [22] developed a computational framework for the establishment of an atomic mobility database directly from the experimental composition–distance profiles based on the pragmatic numerical inverse method [16] and incorporated it into the freely accessible HitDIC (High-Throughput Determination of Interdiffusion Coefficients, https://hitdic.com/, accessed on 17 October 2021, version 2.3.0) software [23]. With this computational framework and HitDIC, the experimental composition–distance profiles, instead of interdiffusion coefficients, can be directly used as the input for the assessment of atomic mobilities and their related uncertainties. Then, for the ternary systems, the complex computational process of interdiffusion coefficients can be avoided, resulting in accuracy and efficiency improvements. One more important advantage lies in that the experimental composition–distance profiles in quaternary and higher-order systems can also be employed to assess the atomic mobility parameters in the target system with the computational framework and HitDIC.

Due to the fact of their good castability and low cost, Mg–Al–Zn (AZ) series alloys are widely used in various fields such as automobile, aerospace, and additive manufacturing [24,25,26,27,28]. Sn, as an important alloying element, is usually introduced to improve the mechanical properties of AZ series alloys [29,30,31]. In order to precisely design the optimal additional amount of Sn in AZ alloys, accurate diffusion coefficients in hcp (hexagonal close-packed) Mg–Al–Sn–Zn alloys are needed. Up to now, the atomic mobilities in the hcp Mg–Al–Zn–Sn quaternary system have only been assessed by Zhong et al. [32] according to the traditional approach. Moreover, the composition–distance profiles in one hcp Mg–Al–Zn–Sn quaternary diffusion couple measured by Bryan et al. [33] can only be used to validate the simulation results but cannot be employed in the optimization process of Zhong et al. [32] due to the limitations of the traditional approach. Furthermore, Zhang et al. [34] measured the new composition–distance profiles of the hcp Mg–Al–Sn ternary system at elevated temperatures recently, which should be considered during the update of the atomic mobility database of the hcp Mg–Al–Sn system.

Consequently, the major objectives of this paper were (*i*) to develop an efficient strategy for the maintenance of an atomic mobility database of multicomponent alloys based on the pragmatic numerical inverse method and HitDIC and (*ii*) to apply the developed efficient strategy to update the atomic mobility database of the hcp Mg–Al–Zn–Sn quaternary system and validate the reliability of the updated atomic mobility database.

## 2. An Effective Strategy to Maintain the CALPHAD Atomic Mobility Database of Multicomponent Systems

**Figure 1:**
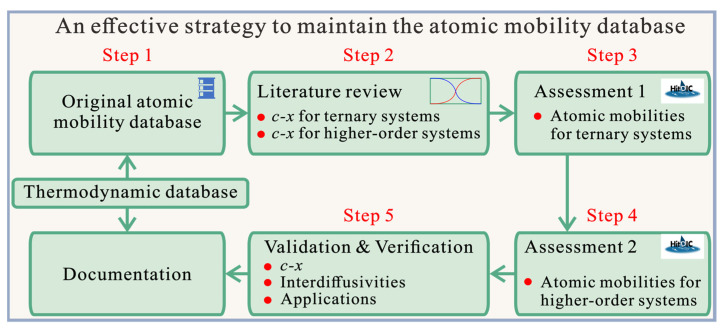
An effective strategy for maintaining the atomic mobility database of multicomponent systems proposed in this work. Here, the “*c*–*x*” represents the “composition–distance”.

Starting from the pragmatic numerical inverse method and HitDIC software, an effective strategy to maintain the atomic mobility database of multicomponent systems is proposed in Figure 1, and it can be separated into the following steps:

1. The original atomic mobility descriptions of the target multicomponent system, together with the thermodynamic descriptions, should be ready or re-constructed according to the corresponding publication(s).
2. A critical review of all the composition–distance profiles of diffusion multiples/couples in ternary and higher-order systems available in the literature should be conducted both before and after the publication/release of the original atomic mobility database.
3. The atomic mobility descriptions in each boundary ternary system should be updated by means of the HitDIC software based on the reviewed composition–distance profiles. It should be noted that the atomic mobility descriptions in all the boundary binaries are fixed during the entire stage. Moreover, the reliability of the updated atomic mobilities should be validated by the experimental composition–distance profiles as well as the evaluated interdiffusion coefficients available in the literature.
4. Based on the updated atomic mobilities of boundary ternary systems, all the composition–distance profiles in the higher-order systems should be input into the HitDIC software to assess the possible interaction parameters in high-order systems. The interaction parameters in higher-order systems are introduced if their addition can really improve the fit to most of the experimental composition profiles. During this step, it should be noted that the interaction parameters of ternary atomic mobilities can be updated if a better fit to the experimental composition profiles in higher-order system can be achieved.
5. One needs to validate the updated atomic mobility database by comprehensively comparing the predicted diffusion properties with the experimental ones in all the related ternary, quaternary, and higher-order systems, verify the updated atomic mobility database by applying real applications if available, and finalize the documentation.

## 3. Literature Review on Diffusion Information in Hcp Mg–Al–Zn–Sn Alloys

In this paper, the atomic mobilities of three boundary binaries (i.e., hcp Mg–Al, Mg–Zn, and Mg–Sn) were directly taken from Zhong et al. [32] and fixed during the subsequent assessment of atomic mobilities in higher-order systems (i.e., hcp Mg–Al–Zn, Mg–Al–Sn, and Mg–Al–Zn–Sn); thus, there was no need to conduct the literature review for those boundary binaries. In the following, all the measured composition–distance profiles in the hcp Mg–Al–Zn, Mg–Al–Sn, and Mg–Al–Zn–Sn systems available in the literature are briefly introduced and are also summarized in Table 1. Moreover, in order to validate the reliability of the subsequently assessed mobilities, the experimental reports on different diffusivities in the hcp Mg–Al–Zn, Mg–Al–Sn, and Mg–Al–Zn–Sn systems were also briefly described as follows.

For the hcp Mg–Al–Zn ternary system, the interdiffusion behaviors in seven groups of diffusion couples at 673 and 723 K were investigated by Kammerer et al. [35]. Thereinto, the composition–distance profiles of four groups (i.e., Mg-9.08Al/Mg-2.55Zn, Mg-0.87Al/Mg-1.12Zn, Mg-9.10Al/Mg-2.03Zn, and Mg-2.27Al/Mg-1.06Zn, in at.%) were reported, while only the diffusion paths were given for the other three groups (i.e., Mg-3Al/Mg-1Zn, Mg-3Al/Mg-0.5Zn, and Mg/Mg-3Al-0.5Zn, in at.%). However, it should be noted that the composition–distance profiles of Mg-0.87Al/Mg-1.12Zn from Kammerer et al. [35] were not reasonable based on the analysis of Wang et al. [36]. In addition, the composition–distance profiles in the AZ91 (Mg-9Al-1Zn, in wt.%)/Mg diffusion couple at 663 and 708 K were also determined by Bryan et al. [33]. But Zhong et al. [32] pointed out that the existence of MgO on the surface of the diffusion couples showed a noticeable effect on the interdiffusion between pure Mg and AZ91 in the work of Bryan et al. [33]. Hence, the composition–distance profiles of three groups (i.e., Mg-9.08Al/Mg-2.55Zn, Mg-9.10Al/Mg-2.03Zn, and Mg-2.27Al/Mg-1.06Zn, in at.%) from Kammerer et al. [35] were employed in the present optimization, while the composition–distance profiles from Bryan et al. [33] as well as the composition–distance profiles of Mg-0.87Al/Mg-1.12Zn and diffusion paths from Kammerer et al. [35] were not.

For the hcp Mg–Al–Sn ternary system, Zhou et al. [37] determined the composition–distance profiles at 673 and 723 K based on the diffusion couple technique. Moreover, the composition–distance profiles for the Mg–Al–Sn ternary diffusion couples at 723, 773, and 823 K were determined by Zhang et al. [34], respectively. The experimental data from both Zhou et al. [37] and Zhang et al. [34] were employed in the present optimization.

As for the hcp Mg–Al–Zn–Sn quaternary system, the composition–distance profiles in one quaternary diffusion couple annealed at 773 K for 250 h were measured by Bryan et al. [33] and thus were considered during the present assessment of the atomic mobilities.

Besides the above experimental information on the composition–distance profiles, there are also some reports on the inter-diffusivities available in the literature. Based on the experimental composition profiles in the hcp Mg–Al–Zn system by Kammerer et al. [35], Wang et al. [36] evaluated the main interdiffusion coefficients (i.e., ${\tilde{D}}_{\mathrm{AlAl}}^{\mathrm{Mg}}$ and ${\tilde{D}}_{\mathrm{ZnZn}}^{\mathrm{Mg}}$) and cross-interdiffusion coefficients (i.e., ${\tilde{D}}_{\mathrm{AlZn}}^{\mathrm{Mg}}$ and ${\tilde{D}}_{\mathrm{ZnAl}}^{\mathrm{Mg}}$) at common intersection points using the Whittle–Green (W–G) method [38]. For the hcp Mg–Al–Sn ternary system, the interdiffusion coefficients (i.e., ${\tilde{D}}_{\mathrm{AlAl}}^{\mathrm{Mg}}$, ${\tilde{D}}_{\mathrm{SnSn}}^{\mathrm{Mg}}$, ${\tilde{D}}_{\mathrm{AlSn}}^{\mathrm{Mg}}$, and ${\tilde{D}}_{\mathrm{SnAl}}^{\mathrm{Mg}}$) at the intersection compositions along diffusion paths were determined by Zhou et al. [37] also using the W–G method. Moreover, the inter-diffusivities of the hcp Mg–Al–Sn system were also determined by Zhang et al. [34] by means of the M–K method. As indicated above, all the related interdiffusion coefficients in ternary systems were not used in the assessment procedure but employed to validate the finally obtained atomic mobilities.

## 4. Results and Discussion

The thermodynamic descriptions for the Mg–Al–Zn–Sn quaternary system from our previous publications [39,40,41] were directly employed in the present work for providing accurate thermodynamic properties. In the following, the atomic mobilities in the hcp Mg–Al–Zn and hcp Mg–Al–Sn ternary systems were first updated by fixing the atomic mobilities in boundary binaries, from which the atomic mobility database in the hcp Mg–Al–Zn–Sn quaternary system was then established.

In the hcp Mg–Al–Zn ternary system, the composition–distance profiles measured by Kammerer [35] (except for those in the Mg-0.87Al/Mg-1.12Zn diffusion couple, in at.%) together with the atomic mobility descriptions of boundary binaries as well as the thermodynamic descriptions were first provided as input in HitDIC software. Subsequently, the initial values of the interaction parameters (i.e., $\Phi_{\mathrm{Al}}^{\mathrm{Mg},\mathrm{Zn}}$ and $\Phi_{\mathrm{Zn}}^{\mathrm{Mg},\mathrm{Al}}$) of the ternary system were automatically set, and the optimization of the two parameters was carried out automatically by the HitDIC software until the best fit between the model-predicted composition–distance profiles and the experimental data was achieved. Finally, the established atomic mobility database of the hcp Mg–Al–Zn ternary system was validated by comparing the predicted diffusion properties with the corresponding experimental data. Moreover, a similar strategy was adopted for the hcp Mg–Al–Sn ternary system.

As for the hcp Mg–Al–Zn–Sn quaternary system, the experimental composition–distance profiles by Bryan et al. [33] together with the updated atomic mobility descriptions of the hcp Mg–Al–Zn and Mg–Al–Sn as well as the thermodynamic descriptions of the hcp Mg–Al–Zn–Sn quaternary systems were first provided as the input in the HitDIC software. Subsequently, the assessment of the interaction parameters in the ternary and/or quaternary systems was automatically performed. It was found that introduction of an interaction parameter (i.e., $\Phi_{\mathrm{Zn}}^{\mathrm{Mg},\mathrm{Sn}}$) can result in the best fit to the experimental data. The finally obtained atomic mobility parameters of the hcp Mg–Al–Zn–Sn quaternary system are summarized in Table 2.

### 4.1. Hcp Mg–Al–Zn Ternary System

**Figure 2:**
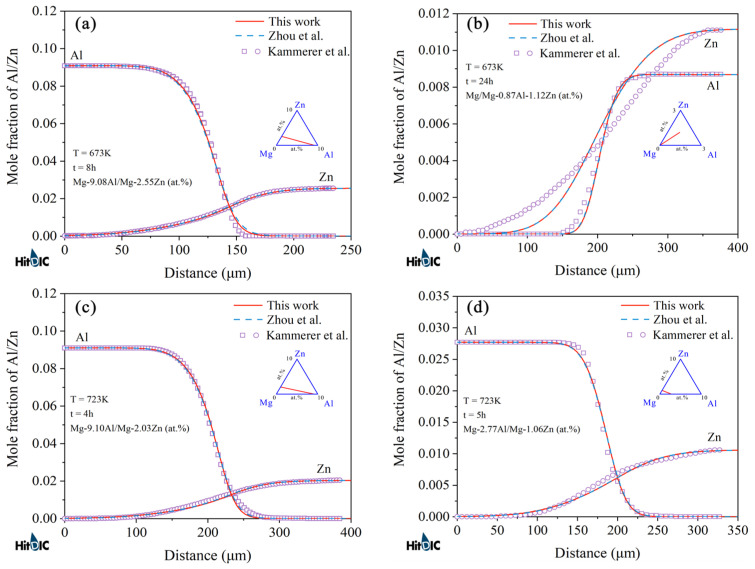
Model-predicted composition–distance profiles of different hcp Mg–Al–Zn diffusion couples annealed at (**a**) 673 K for 8 h, (**b**) 673 K for 24 h, (**c**) 723 K for 4 h, and (**d**) 723 K for 5 h, due to the present atomic mobilities (solid lines), compared with those of Zhong et al. [32] (dashed lines) and the experimental data [35] (in symbols).

**Figure 3:**
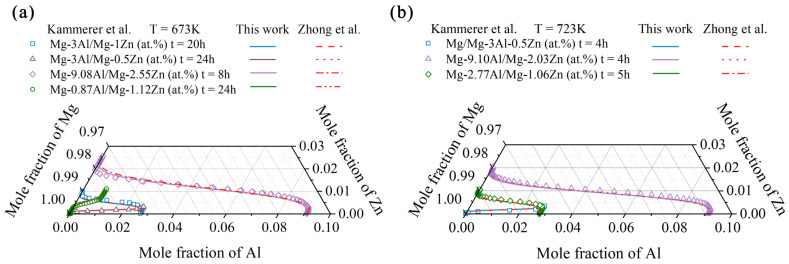
Model-predicted diffusion paths in the hcp Mg–Al–Zn system at (**a**) 673 and (**b**) 723 K due to the present atomic mobilities (solid lines) compared with those of Zhong et al. [32] (dashed lines) and the experimental data [35] (in symbols).

The model-predicted composition–distance profiles of four diffusion couples (i.e., Mg-9.08Al/Mg-2.55Zn at 673 K for 8 h, Mg/Mg-0.87Al-1.12Zn at 673 K for 24 h, Mg-9.10Al/Mg-2.03Zn at 723 K for 4 h, and Mg-2.77Al/Mg-1.06Zn at 723 K for 5 h, in at.%) according to the present atomic mobilities (solid lines) are displayed in Figure 2, compared with the corresponding experimental data (in symbols) by Kammerer et al. [35]. The model-predicted results by Zhong et al. [32] are also superimposed as dashed lines in the figure for direct comparison with the present results. Without specification, all the model-predicted results of Zhong et al. [32] are taken exactly from their original publication. As can be seen in Figure 2, the predicted results from the present work are consistent with those from Zhong et al. [32], and both predicted results are in good agreement with the experimental composition–distance profiles [35], expect for Figure 2b. As shown in Figure 2b, a large deviation between the model-predicted composition–distance profile of Zn and the experimental ones can be observed. This fact is quite normal because the composition–distance profiles of Mg/Mg-0.87Al-1.12Zn at 673 K for 24 h are not reasonable based on the suggestion by Wang et al. [36] and, thus, were not employed in the present optimization. Furthermore, the model-predicted diffusion paths at 673 and 723 K, based on the present atomic mobilities together with those by Zhong et al. [32], are shown in Figure 3 compared with the experimental data [35]. The diffusion paths predicted according to the present atomic mobilities are in very good agreement with the experimental data [35] and also the ones by Zhong et al. [32]. Moreover, the comparison between the model-predicted composition–distance profiles due to the present atomic mobilities and the experimental data by Bryan et al. [33] as well as those by Zhong et al. [32] are displayed in the Supplementary Materials for readers’ reference. As can be seen Figure S1, certain deviations exist between the simulated composition profiles of Al/Zn and the experimental data. This is because MgO exits on the surface of the diffusion couples, as pointed out by Zhong et al. [32], and hinders the diffusion of both Al and Zn. Thus, the composition–distance profiles of Bryan et al. [33] were not considered in the present update of atomic mobilities.

**Figure 4:**
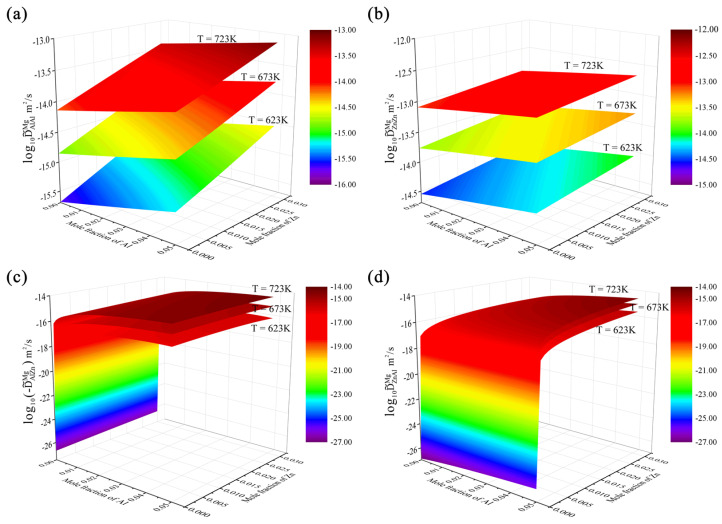
Model-predicted composition-dependent inter-diffusivities of (**a**) ${\tilde{D}}_{\mathrm{AlAl}}^{\mathrm{Mg}}$, (**b**) ${\tilde{D}}_{\mathrm{ZnZn}}^{\mathrm{Mg}}$, (**c**) ${\tilde{D}}_{\mathrm{AlZn}}^{\mathrm{Mg}}$, and (**d**) ${\tilde{D}}_{\mathrm{ZnAl}}^{\mathrm{Mg}}$ over the composition range of 0–5.0 at.% Al and 0–3.0 at.% Zn at 623, 673, and 723 K according to the present atomic mobilities together with the thermodynamic descriptions [42].

According to the presently updated atomic mobility descriptions together with the thermodynamic descriptions [42], the interdiffusion coefficients of the hcp Mg–Al–Zn system over the composition range of 0–5.0 at.% Al and 0–3.0 at.% Zn at 623, 673, and 723 K are predicted in Figure 4. Figure 4a,b show the calculated main interdiffusion coefficients, ${\tilde{D}}_{\mathrm{AlAl}}^{\mathrm{Mg}}$ and ${\tilde{D}}_{\mathrm{ZnZn}}^{\mathrm{Mg}}$, in three-dimensional space, respectively. As shown in Figure 4a,b, ${\tilde{D}}_{\mathrm{ZnZn}}^{\mathrm{Mg}}$ was larger than ${\tilde{D}}_{\mathrm{AlAl}}^{\mathrm{Mg}}$ at the same temperature by approximately one order of magnitude, which means that the diffusion rate of Zn in hcp Mg–Al–Zn alloys is faster than that of Al. Moreover, it can be observed that both ${\tilde{D}}_{\mathrm{AlAl}}^{\mathrm{Mg}}$ and ${\tilde{D}}_{\mathrm{ZnZn}}^{\mathrm{Mg}}$ increased with the increase in temperature and concentrations of both Al and Zn. Figure 4c displays variations in the cross-interdiffusion coefficient ${\tilde{D}}_{\mathrm{AlZn}}^{\mathrm{Mg}}$ along with the concentrations of Al and Zn. It should be noted that the predicted ${\tilde{D}}_{\mathrm{AlZn}}^{\mathrm{Mg}}$ over wide composition and temperature range is negative. Hence, the $\log_{10}(-{\tilde{D}}_{\mathrm{AlZn}}^{\mathrm{Mg}})$ was adopted for the label of ordinate of Figure 4c in order to facilitate the analysis. The sign of cross-interdiffusion coefficients had been analyzed in detail by Liu et al. [43] in terms of thermodynamics. According to Liu et al. [43], the cross-interdiffusion coefficient ${\tilde{D}}_{\mathrm{AlZn}}^{\mathrm{Mg}}$ in the hcp Mg–Al–Zn ternary system can be expressed as follows:

(1) $${\tilde{D}}_{\mathrm{AlZn}}^{\mathrm{Mg}}=\left[{\left(1-x_{Al}\right)}^{2}x_{Al}M_{Al}+x_{Al}^{2}x_{Zn}M_{Zn}+x_{Al}^{2}x_{Mg}M_{Mg}\right]\frac{\partial (\mu_{Al}-\mu_{Mg})}{\partial x_{Zn}}$$

where *M_Al_*, *M_Zn_*, and *M_Mg_* are the atomic mobilities for Al, Zn, and Mg, respectively. *x_Al_*, *x_Zn_*, and *x_Mg_* are the mole fractions for Al, Zn, and Mg, respectively. *μ_Al_* and *μ_Mg_* represent the chemical potentials of Al and Mg, respectively. Because the term before $\partial (\mu_{Al}-\mu_{Mg})/\partial x_{Zn}$ in Equation (1) is positive, the negative sign of ${\tilde{D}}_{\mathrm{AlZn}}^{\mathrm{Mg}}$ is determined by the $\partial (\mu_{Al}-\mu_{Mg})/\partial x_{Zn}$. As can be seen in Figure 4c, the ${\tilde{D}}_{\mathrm{AlZn}}^{\mathrm{Mg}}$ was lower than the main interdiffusion coefficients, ${\tilde{D}}_{\mathrm{AlAl}}^{\mathrm{Mg}}$ and ${\tilde{D}}_{\mathrm{ZnZn}}^{\mathrm{Mg}}$, at the same temperature by approximately one to two orders of magnitude. Moreover, the ${\tilde{D}}_{\mathrm{AlZn}}^{\mathrm{Mg}}$ increased with the increase in temperature and Zn concentration. While ${\tilde{D}}_{\mathrm{AlZn}}^{\mathrm{Mg}}$ increased rapidly as the Al concentration increased in the region where the Al concentration was close to zero, but then increased slowly with the further increase in Al concentration. Furthermore, it is interesting to see in Figure 4c that the cross-interdiffusion coefficients ${\tilde{D}}_{\mathrm{AlZn}}^{\mathrm{Mg}}$ at 623, 673, and 723 K were all approaching zero as the concentration of Al approaches zero. It should be noted that such an interesting phenomenon is reasonable and can be obviously proved by Equation (1). The relationship between the cross-interdiffusion coefficient ${\tilde{D}}_{\mathrm{ZnAl}}^{\mathrm{Mg}}$ and concentrations of both Al and Zn is displayed in Figure 4d. Different from ${\tilde{D}}_{\mathrm{AlZn}}^{\mathrm{Mg}}$, the presently predicted ${\tilde{D}}_{\mathrm{ZnAl}}^{\mathrm{Mg}}$ is positive, and can be expressed as the following equation similar to Equation (1):

(2) $${\tilde{D}}_{\mathrm{ZnAl}}^{\mathrm{Mg}}=\left[{\left(1-x_{Zn}\right)}^{2}x_{Zn}M_{Zn}+x_{Zn}^{2}x_{Al}M_{Al}+x_{Zn}^{2}x_{Mg}M_{Mg}\right]\frac{\partial (\mu_{Zn}-\mu_{Mg})}{\partial x_{Al}}$$

According to Equation (2), the positive sign of ${\tilde{D}}_{\mathrm{ZnAl}}^{\mathrm{Mg}}$ is due to the positive $\partial (\mu_{Zn}-\mu_{Mg})/\partial x_{Al}$. As shown in Figure 4d, the value of ${\tilde{D}}_{\mathrm{ZnAl}}^{\mathrm{Mg}}$ is in the same order of the absolute one of ${\tilde{D}}_{\mathrm{AlZn}}^{\mathrm{Mg}}$, but lower than the main interdiffusion coefficients, ${\tilde{D}}_{\mathrm{AlAl}}^{\mathrm{Mg}}$ and ${\tilde{D}}_{\mathrm{ZnZn}}^{\mathrm{Mg}}$. In addition, the ${\tilde{D}}_{\mathrm{ZnAl}}^{\mathrm{Mg}}$ increased with the increase in temperature and Al concentration, while the ${\tilde{D}}_{\mathrm{ZnAl}}^{\mathrm{Mg}}$ increased rapidly in the region where the Zn concentration was close to zero, and then increased slowly with the further increase of Zn. Moreover, an interesting phenomenon can also be found with the cross-interdiffusion coefficients ${\tilde{D}}_{\mathrm{ZnAl}}^{\mathrm{Mg}}$ at 623, 673, and 723 K all approaching zero as the concentration of Zn approached zero.

**Figure 5:**
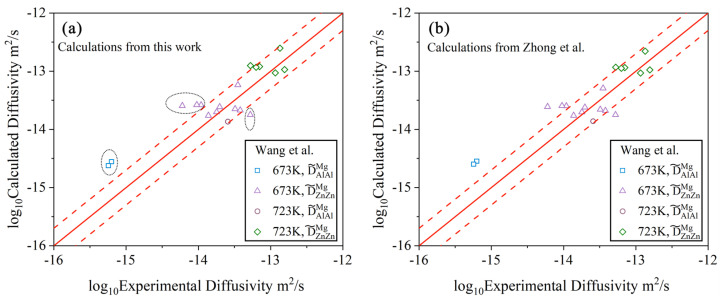
Model-predicted main inter-diffusivities in the hcp Mg–Al–Zn system due to (**a**) the present atomic mobilities and (**b**) Zhong et al. [32] at 673 and 723 K compared with the experimental data [36]. Along the diagonal lines, the model-predicted values are exactly equal to the experimental ones. The dashed lines represent the interdiffusion coefficients multiplied by a pre-factor of 2 or 0.5.

To further illustrate the reliability of the presently updated atomic mobilities, the calculated main inter-diffusivities according to the present work are compared with the determined ones by Wang et al. [36] in Figure 5a. Along the diagonal lines, the model-predicted values are exactly equal to the experimental ones. The region of empirical errors for inter-diffusivities is constructed by the two dashed lines that represent the interdiffusion coefficients multiplied with a pre-factor of 2 or 0.5, respectively, according to the suggestion in [44]. A similar plot was also made in Figure 5b between the calculated main inter-diffusivities by Zhong et al. [32] and the ones determined by Wang et al. [36]. Based on the comparison in Figure 5a,b, it can be found that the calculated main interdiffusion coefficients from the present work are consistent with those of Zhong et al. [32], and the calculated main interdiffusion coefficients in both the present work and Zhong et al. [32] agree well with all the experimental data (within the dashed lines), expected for 6 values marked by black circles in the figure. It should be noted that those 6 points marked by black circles were determined by Wang et al. [36] based on three diffusion couples (i.e., Mg-3Al/Mg-1Zn, Mg-3Al/Mg-0.5Zn, and Mg-0.87Al/Mg-1.12Zn) from Kammerer et al. [35] of which the composition–distance profiles were not employed in the present optimization because the original experimental data were either unreasonable (i.e., Mg-0.87Al/Mg-1.12Zn) or not provided (i.e., Mg-3Al/Mg-1Zn and Mg-3Al/Mg-0.5Zn) according to the original publications.

Based on the above analysis, the presently updated atomic mobilities of the hcp Mg–Al–Zn based on the newly proposed strategy are reliable and can give as good fit to all the experimental properties as of the recent publication [32] using the traditional approach.

### 4.2. Hcp Mg–Al–Sn Ternary System

**Figure 6:**
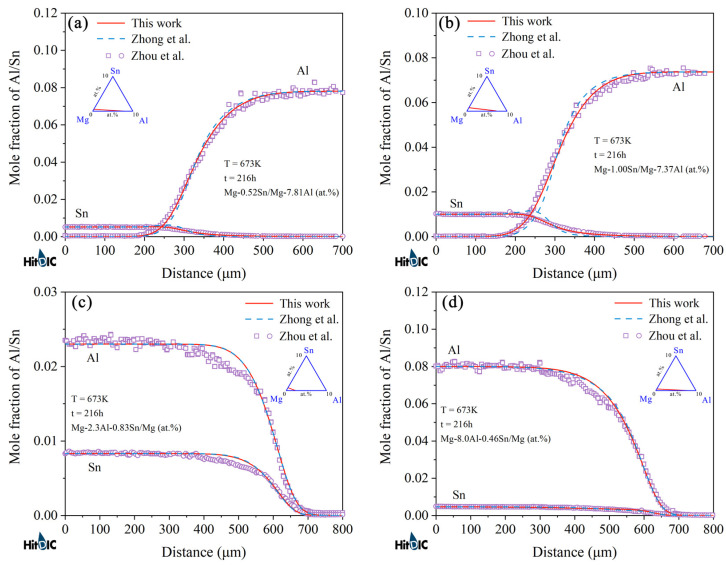
(**a**–**d**) Model-predicted composition–distance profiles of the different hcp Mg–Al–Sn diffusion couples annealed at 673 K for 216 h from the present atomic mobilities (solid lines) compared with those of Zhong et al. [32] (dashed lines) and the experimental data [37] (in symbols).

**Figure 7:**
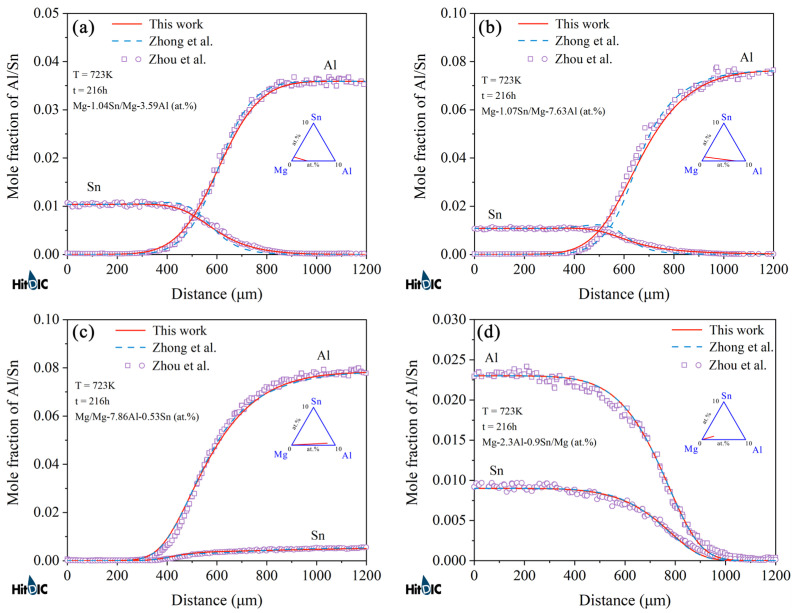
(**a**–**d**) Model-predicted composition–distance profiles of the different hcp Mg–Al–Sn diffusion couples annealed at 723 K for 216 h from the present atomic mobilities (solid lines) compared with those of Zhong et al. [32] (dashed lines) and the experimental data [37] (in symbols).

**Figure 8:**
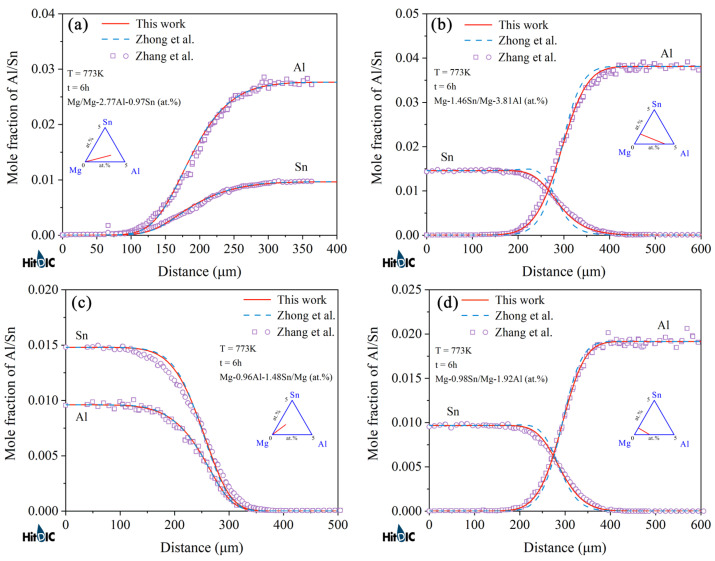
(**a**–**d**) Model-predicted composition–distance profiles of the different hcp Mg–Al–Sn diffusion couples annealed at 773 K for 6 h from the present atomic mobilities (solid lines) compared with those of Zhong et al. [32] (dashed lines) and the experimental data [34] (in symbols).

**Figure 9:**
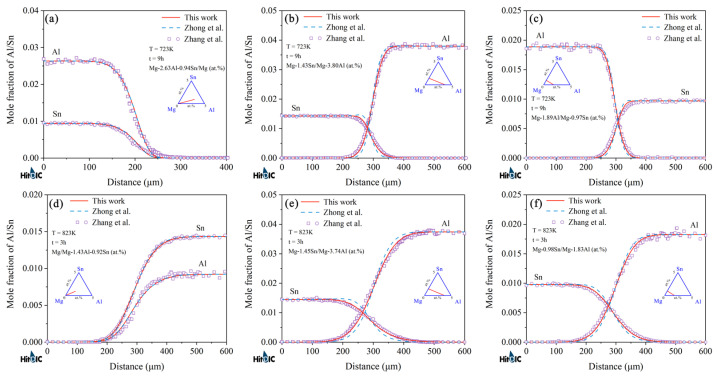
Model-predicted composition–distance profiles of the different hcp Mg–Al–Sn diffusion couples annealed at (**a**–**c**) 723 K for 9 h and (**d**–**f**) 823 K for 3 h from the present atomic mobilities (solid lines) compared with these of Zhong et al. [32] (dashed lines) and the experimental data [34] (in symbols).

**Figure 10:**
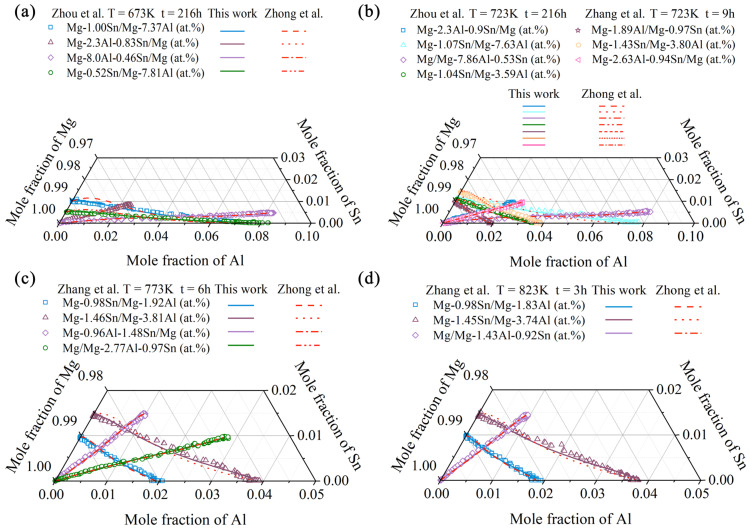
Model-predicted diffusion paths in the hcp Mg–Al–Sn system at (**a**) 673 K for 216 h, (**b**) 723 K for 216 and 9 h, (**c**) 773 K for 6 h, and (**d**) 823 K for 3 h from the present mobilities (solid lines) compared with those of Zhong et al. [32] (dashed lines) and the experimental data [34,37] (in symbols).

Figure 6 and Figure 7 display the model-predicted composition–distance profiles of eight diffusion couples (i.e., Mg-0.52Sn/Mg-7.81Al, Mg-1.00Sn/Mg-7.37Al, Mg-2.30Al-0.83Sn/Mg, and Mg-8.00Al-0.46Sn/Mg, annealed at 673 K for 216 h, in at.%; Mg-1.04Sn/Mg-3.59Al, Mg-1.07Sn/Mg-7.63Al, Mg/Mg-7.86Al-0.53Sn, and Mg-2.3Al-0.9Sn/Mg, annealed at 723 K for 216 h, in at.%) from the present work (solid lines) compared with the experimental data (in symbols) by Zhou et al. [37]. The model-predicted results according to Zhong et al. [32] (dashed lines) are also superimposed in the figure for direct comparison. Figure 8 and Figure 9 also show the model-predicted composition–distance profiles of 10 diffusion couples (i.e., Mg/Mg-2.77Al-0.97Sn, Mg-1.46Sn/Mg-3.81Al, Mg-0.96Al-1.48Sn/Mg, and Mg-0.98Sn/Mg-1.92Al, annealed at 773 K for 6 h, in at.%; Mg-2.63Al-0.94Sn/Mg, Mg-1.43Sn/Mg-3.80Al, and Mg-1.89Al/Mg-0.97Sn, annealed at 723 K for 9 h, in at.%; Mg/Mg-1.43Al-0.92Sn, Mg-1.45Sn/Mg-3.74Al, and Mg-0.98Sn/Mg-1.83Al, annealed at 823 K for 3 h, in at.%) according to the present atomic mobilities and also those from Zhong et al. [32] compared with the experimental data by Zhang et al. [34]. As can be seen in Figure 6, Figure 7, Figure 8 and Figure 9, the model-predicted composition–distance profiles according to the present work are in better agreement with the experimental data by Zhou et al. [37] and Zhang et al. [34] than the model-predicted ones due from Zhong et al. [32], especially in the figures, i.e., Figure 6a,b, Figure 7a,b, Figure 8b,d and Figure 9b,c,e,f. Furthermore, the model-predicted diffusion paths at 673, 723, 773, and 823 K, based on the presently updated atomic mobilities and also those by Zhong et al. [32], are displayed in Figure 10 compared with the experimental data from Zhou et al. [37] and Zhang et al. [34]. As can be seen in Figure 10, the model-predicted diffusion paths by the present work again agree better with the experimental data [34,37] than the model-predicted ones by Zhong et al. [32].

**Figure 11:**
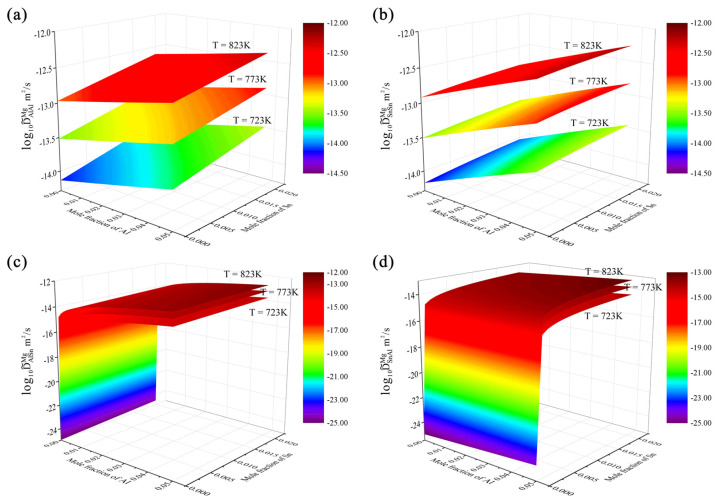
Model-predicted composition-dependent inter-diffusivities of (**a**) ${\tilde{D}}_{\mathrm{AlAl}}^{\mathrm{Mg}}$, (**b**) ${\tilde{D}}_{\mathrm{SnSn}}^{\mathrm{Mg}}$, (**c**) ${\tilde{D}}_{\mathrm{AlSn}}^{\mathrm{Mg}}$, and (**d**) ${\tilde{D}}_{\mathrm{SnAl}}^{\mathrm{Mg}}$ over the composition range of 0–5.0 at.% Al and 0–2.0 at.% Sn at 723, 773, and 823 K according to the present atomic mobilities together with the thermodynamic descriptions [39].

**Figure 12:**
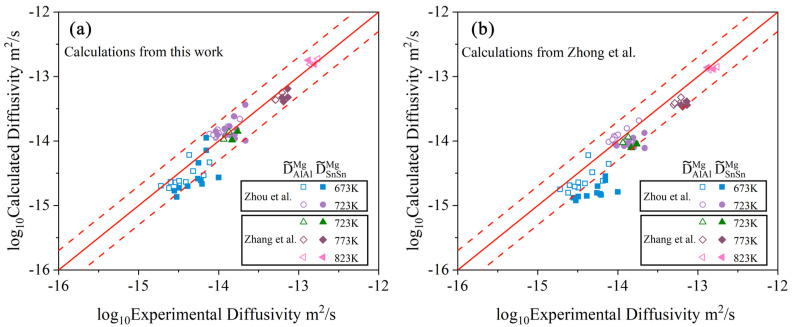
Model-predicted main inter-diffusivities in the hcp Mg–Al–Sn system due to (**a**) the present atomic mobilities and (**b**) Zhong et al. [32] at 673, 723, 773, and 823 K compared with the experimental data [34,37]. Along the diagonal lines, the model-predicted values are exactly equal to the experimental ones. The dashed lines represent the interdiffusion coefficients multiplied with a pre-factor of 2 or 0.5.

Based on the updated atomic mobility descriptions by the present work together with the thermodynamic descriptions [39], the inter-diffusivities of the hcp Mg–Al–Sn system over the composition range of 0–5.0 at.% Al and 0–2.0 at.% Sn at 723, 773, and 823 K are predicted in Figure 11. Similar to the hcp Mg–Al–Zn system, the interdiffusion coefficients of the hcp Mg–Al–Sn system were also processed with a logarithm. As shown in Figure 11a,b, the main interdiffusion coefficient ${\tilde{D}}_{\mathrm{AlAl}}^{\mathrm{Mg}}$ was in the same order of magnitude as the ${\tilde{D}}_{\mathrm{SnSn}}^{\mathrm{Mg}}$ at the same temperature. Besides, both ${\tilde{D}}_{\mathrm{AlAl}}^{\mathrm{Mg}}$ and ${\tilde{D}}_{\mathrm{SnSn}}^{\mathrm{Mg}}$ increased with the increase in temperature and concentrations of Al and Sn. Figure 11c,d show that the cross-interdiffusion coefficients, ${\tilde{D}}_{\mathrm{AlSn}}^{\mathrm{Mg}}$ and ${\tilde{D}}_{\mathrm{SnAl}}^{\mathrm{Mg}}$, varied apparently along with the concentrations of Al and Sn. Similar to Equations (1) and (2), ${\tilde{D}}_{\mathrm{AlSn}}^{\mathrm{Mg}}$ and ${\tilde{D}}_{\mathrm{SnAl}}^{\mathrm{Mg}}$ can be expressed as:

(3) $${\tilde{D}}_{\mathrm{AlSn}}^{\mathrm{Mg}}=\left[{\left(1-x_{Al}\right)}^{2}x_{Al}M_{Al}+x_{Al}^{2}x_{Sn}M_{Sn}+x_{Al}^{2}x_{Mg}M_{Mg}\right]\frac{\partial (\mu_{Al}-\mu_{Mg})}{\partial x_{Sn}}$$

(4) $${\tilde{D}}_{\mathrm{SnAl}}^{\mathrm{Mg}}=\left[{\left(1-x_{Sn}\right)}^{2}x_{Sn}M_{Sn}+x_{Sn}^{2}x_{Al}M_{Al}+x_{Sn}^{2}x_{Mg}M_{Mg}\right]\frac{\partial (\mu_{Sn}-\mu_{Mg})}{\partial x_{Al}}$$

where *M_Al_*, *M_Sn_*, and *M_Mg_* are the atomic mobilities for Al, Sn, and Mg, respectively. *x_Al_*, *x_Sn_*, and *x_Mg_* are the mole fractions for Al, Sn, and Mg, respectively. *μ_Al_*, *μ_Sn_*, and *μ_Mg_* represent the chemical potentials of Al, Sn, and Mg, respectively. Here, it should be noted that the signs of ${\tilde{D}}_{\mathrm{AlSn}}^{\mathrm{Mg}}$ and ${\tilde{D}}_{\mathrm{SnAl}}^{\mathrm{Mg}}$ are positive, which are determined by the terms $\partial (\mu_{Al}-\mu_{Mg})/\partial x_{Sn}$ and $\partial (\mu_{Sn}-\mu_{Mg})/\partial x_{Al}$, respectively. As can be seen in Figure 11c,d, the cross-interdiffusion coefficient ${\tilde{D}}_{\mathrm{AlSn}}^{\mathrm{Mg}}$ was in the same order of the main interdiffusion coefficients, ${\tilde{D}}_{\mathrm{AlAl}}^{\mathrm{Mg}}$ and ${\tilde{D}}_{\mathrm{SnSn}}^{\mathrm{Mg}}$, at the same temperature, while the cross-interdiffusion coefficient ${\tilde{D}}_{\mathrm{SnAl}}^{\mathrm{Mg}}$ was lower than the main interdiffusion coefficients, ${\tilde{D}}_{\mathrm{AlAl}}^{\mathrm{Mg}}$ and ${\tilde{D}}_{\mathrm{SnSn}}^{\mathrm{Mg}}$, by approximately one order of magnitude. Moreover, ${\tilde{D}}_{\mathrm{AlSn}}^{\mathrm{Mg}}$ increased with the increase in temperature and Sn concentration, while ${\tilde{D}}_{\mathrm{AlSn}}^{\mathrm{Mg}}$ increased rapidly in the region where the Al concentration was close to zero, and then increased slowly with the further increase in Al. As the concentration of Al (Sn) approached zero, the cross-interdiffusion coefficient, ${\tilde{D}}_{\mathrm{AlSn}}^{\mathrm{Mg}}$ (${\tilde{D}}_{\mathrm{SnAl}}^{\mathrm{Mg}}$) at 723, 773, and 823 K were all approaching zero, which can be reasonably explained by Equations (3) and (4). Figure 12a,b respectively show the calculated main interdiffusion coefficients according to the present atomic mobilities and those of Zhong et al. [32], compared with the experimental data [34,37]. Along the diagonal lines, the model-predicted values are exactly equal to the experimental ones. The two dashed lines represent the interdiffusion coefficients multiplied by a pre-factor of 2 or 0.5, respectively. A comparison between Figure 12a,b clearly indicates that the calculated interdiffusion coefficients from the present work can reproduce more experimental data than those by Zhong et al. [32].

Based on the above comprehensive comparison among the model-predicted results from the present work, the ones by Zhong et al. [32], and the experimental data [34,37], the reliability of the atomic mobility descriptions of the hcp Mg–Al–Sn system was significantly improved by using the newly proposed strategy compared with the traditional approach. The major reason lies in that although 18 groups of diffusion couples were investigated by Zhou et al. [37] and Zhang et al. [34], only very scattered experimental interdiffusion coefficients at the intersection compositions of diffusion paths can be determined by the traditional methods and then utilized in the traditional optimization process, which may lead to the lower accuracy of the obtained atomic mobility descriptions. By contrast, all 18 groups of composition–distance profiles can be employed in the optimization process using the new strategy based on HitDIC, which can largely improve the reliability of the atomic mobility descriptions.

### 4.3. Hcp Mg–Al–Zn–Sn Quaternary System

**Figure 13:**
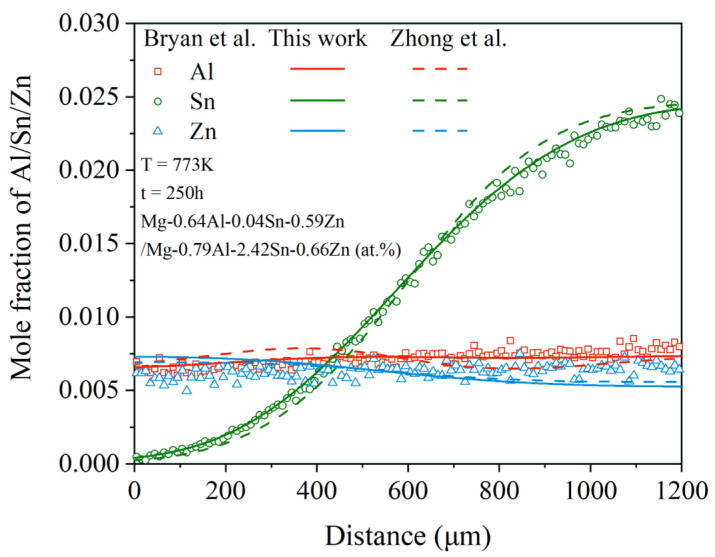
Model-predicted composition–distance profiles of the only quaternary diffusion couple in quaternary system, Mg-0.64Al-0.04Sn-0.59Zn/Mg-0.79Al-2.42Sn-0.66Zn, annealed at 773 K for 250 h from the present atomic mobilities (solid lines) compared with these of Zhong et al. [32] (dashed lines) and the experimental data [33] (in symbols).

Figure 13 displays the comparison between the model-predicted composition–distance profiles of the only quaternary diffusion couple, Mg-0.64Al-0.04Sn-0.59Zn/Mg-0.79Al-2.42Sn-0.66Zn, annealed at 773 K for 250 h due to the present atomic mobilities (solid lines) and the experimental data (in symbols) by Bryan et al. [33]. The model-predicted composition–distance profiles from Zhong et al. [32] (dashed lines) are also superimposed in the figure for direct comparison. As can be seen in Figure 13, the model-predicted composition–distance profiles of Sn and Al in the present work show much better agreement with the experimental data of Bryan et al. [33], compared with the results from Zhong et al. [32]. The model-predicted composition–distance curve of Zn from both the present work and Zhong et al. [32] slightly deviate from the experimental data of Bryan et al. [33]. It should be noted that the difference in the Zn concentration in both end alloys was only 0.07 at.%, which may cause large difficulties in the accurate experimental measurement of Zn concentration.

Based on the above analysis, a real improvement in the reproduction of the experimental data was achieved by the present work compared with the results of Zhong et al. [32], even though only one more quaternary diffusion couple was included in the present work. It is anticipated that the reliability of the atomic mobilities in the hcp Mg–Al–Zn–Sn quaternary system can be further improved by using the newly proposed strategy if more experimental composition profiles in the quaternary Mg–Al–Zn–Sn system are available. By contrast, the reliability of atomic mobilities in the hcp Mg–Al–Zn–Sn quaternary system cannot be improved based on the traditional method, no matter whether the experimental data for Mg–Al–Zn–Sn system are sufficient.

**Figure 14:**
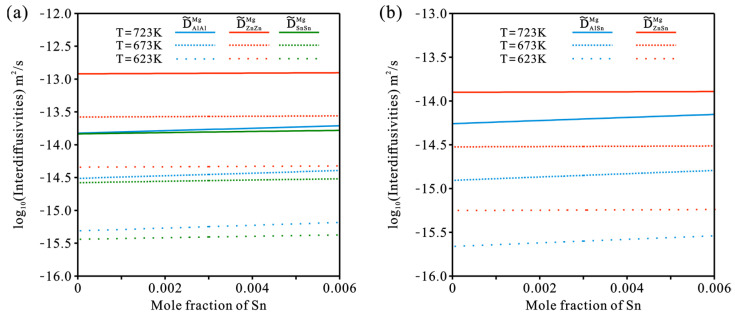
Model-predicted component-dependent inter-diffusivities of (**a**) ${\tilde{D}}_{\mathrm{AlAl}}^{\mathrm{Mg}}$, ${\tilde{D}}_{\mathrm{ZnZn}}^{\mathrm{Mg}}$, and ${\tilde{D}}_{\mathrm{SnSn}}^{\mathrm{Mg}}$ and (**b**) ${\tilde{D}}_{\mathrm{AlSn}}^{\mathrm{Mg}}$ and ${\tilde{D}}_{\mathrm{ZnSn}}^{\mathrm{Mg}}$ over a composition range of 2.4 at.% Al, 0.56 at.% Zn, and 0–0.3 at.% Sn at 623, 673, and 723 K according to the present atomic mobilities together with the thermodynamic descriptions [40].

To illustrate the influence of Sn concentration and temperature on the inter0diffusivities of the hcp Mg–Al–Zn–Sn quaternary system, the matrix (α-Mg) phase with an average composition of 2.4 at.% Al and 0.56 at.% Zn in as-cast AZT640 (Mg-6Al-4Zn-0.6Sn, in wt.%), according to Dong et al. [45], was chosen as the target in the present work. According to the presently updated atomic mobility descriptions together with the thermodynamic descriptions [40], the interdiffusion coefficients of the Mg–Al–Zn–Sn quaternary system over a compositions range of 2.4 at.% Al, 0.56 at.% Zn, and 0–0.6 at.% Sn at 623, 673, and 723 K were predicted in Figure 14. As can be seen in Figure 14a, the ${\tilde{D}}_{\mathrm{AlAl}}^{\mathrm{Mg}}$ and ${\tilde{D}}_{\mathrm{SnSn}}^{\mathrm{Mg}}$ were quite close to each other and lower than ${\tilde{D}}_{\mathrm{ZnZn}}^{\mathrm{Mg}}$ by approximately one order of magnitude. Moreover, the ${\tilde{D}}_{\mathrm{AlAl}}^{\mathrm{Mg}}$ and ${\tilde{D}}_{\mathrm{SnSn}}^{\mathrm{Mg}}$ increased with the increase in temperature and Sn concentration, while ${\tilde{D}}_{\mathrm{ZnZn}}^{\mathrm{Mg}}$ increased with the increase in temperature and kept nearly constant with the increment in Sn concentration. Figure 14b displays the variations in cross-interdiffusion coefficients, ${\tilde{D}}_{\mathrm{AlSn}}^{\mathrm{Mg}}$ and ${\tilde{D}}_{\mathrm{ZnSn}}^{\mathrm{Mg}}$, with Sn concentration. ${\tilde{D}}_{\mathrm{ZnSn}}^{\mathrm{Mg}}$ was larger than ${\tilde{D}}_{\mathrm{AlSn}}^{\mathrm{Mg}}$ but lower than the main interdiffusion coefficients, ${\tilde{D}}_{\mathrm{AlAl}}^{\mathrm{Mg}}$ and ${\tilde{D}}_{\mathrm{SnSn}}^{\mathrm{Mg}}$. In addition, ${\tilde{D}}_{\mathrm{ZnSn}}^{\mathrm{Mg}}$ increased with the increase in temperature but kept nearly constant with the increment in Sn concentration, while ${\tilde{D}}_{\mathrm{AlSn}}^{\mathrm{Mg}}$ rose with the increase in temperature and Sn concentration.

## 5. Conclusions

- A general and effective strategy for the maintenance of the CALPHAD atomic mobility database of multicomponent systems was developed based on the pragmatic numerical inverse method and HitDIC software;
- Following the newly proposed strategy, the atomic mobility descriptions of the hcp Mg–Al–Zn and Mg–Al–Sn ternary systems were updated based on the experimental composition profiles in the respective ternary systems. It was found that the presently updated atomic mobilities of the hcp Mg–Al–Zn system provided a good fit for all of the experimental diffusion properties as did the previous assessment [32] using the traditional approach, while the presently updated atomic mobilities of the hcp Mg–Al–Sn system showed better agreement with the experimental diffusion properties than the previous assessment [32] using the traditional approach. Moreover, the variation trend of inter-diffusivities of the hcp Mg–Al–Zn and Mg–Al–Sn systems with the temperature and solute (i.e., Al, Zn, and Sn) concentrations was also fully analyzed;
- Based on the updated atomic mobility descriptions of the hcp Mg–Al–Zn and Mg–Al–Sn systems, together with only one set of composition–distance profiles, the atomic mobility descriptions of the hcp Mg–Al–Zn–Sn quaternary system were further updated following the newly proposed strategy. A real improvement in the reproduction of experimental data was achieved by the present work compared with the previous assessment. Furthermore, the influence of Sn concentration and temperature on the inter-diffusivities of the hcp Mg–Al–Zn–Sn quaternary alloys was also illustrated;
- It is anticipated that the presently proposed strategy can serve as a standard for maintaining the CALPHAD atomic mobility database of different multicomponent systems.

## Figures and Tables

**Table 1:** List of the composition–distance profiles of different hcp_A3 Mg–Al–Zn, Mg–Al–Sn, and Mg–Al–Zn–Sn alloys available in the literature.

Type of Diffusion Couple (in at.%)	Diffusion Temperature (K)	Diffusion Time (h)	References	Code
Mg–Al–Zn ternary system				
Mg-9.08Al/Mg-2.55Zn	673	8	[35]	**▲**
Mg/Mg-0.87Al-1.12Zn		24		**△**
Mg-3Al/Mg-1Zn		20		**△**
Mg-3Al/Mg-0.5Zn		24		**△**
Mg-9.10Al/Mg-2.03Zn	723	4	[35]	**▲**
Mg-2.77Al/Mg-1.06Zn		5		**▲**
Mg/Mg-3Al-0.5Zn		4		**△**
Mg/Mg-8.41Al-0.45Zn	663	144	[33]	**△**
Mg/Mg-8.50Al-0.41Zn	708	144	[33]	**△**
Mg–Al–Sn ternary system				
Mg-0.52Sn/Mg-7.81Al	673	216	[37]	**▲**
Mg-1.00Sn/Mg-7.37Al		216		**▲**
Mg-2.30Al-0.83Sn/Mg		216		**▲**
Mg-8.00Al-0.46Sn/Mg		216		**▲**
Mg-1.04Sn/Mg-3.59Al	723	216	[37]	**▲**
Mg-1.07Sn/Mg-7.63Al		216		**▲**
Mg/Mg-7.86Al-0.53Sn		216		**▲**
Mg-2.3Al-0.9Sn/Mg		216		**▲**
Mg-2.63Al-0.94Sn/Mg	723	9	[34]	**▲**
Mg-1.43Sn/Mg-3.80Al		9		**▲**
Mg-1.89Al/Mg-0.97Sn		9		**▲**
Mg/Mg-2.77Al-0.97Sn	773	6	[34]	**▲**
Mg-1.46Sn/Mg-3.81Al		6		**▲**
Mg-0.96Al-1.48Sn/Mg		6		**▲**
Mg-0.98Sn/Mg-1.92Al		6		**▲**
Mg/Mg-1.43Al-0.92Sn	823	3	[34]	**▲**
Mg-1.45Sn/Mg-3.74Al		3		**▲**
Mg-0.98Sn/Mg-1.83Al		3		**▲**
Mg–Al–Zn–Sn quaternary system				
Mg-0.64Al-0.04Sn-0.59Zn/Mg-0.79Al-2.42Sn-0.66Zn	773	250	[33]	**▲**

▲, used in the optimization process; △, only used for comparison.

**Table 2:** List of the atomic mobility parameters of hcp the Mg–Al–Zn–Sn system assessed in the present work together with those taken in the literature [32].

Mobility	Parameters	References
Mobility of Mg	$\Phi_{\mathrm{Mg}}^{\mathrm{Mg}}$ = −125,748.3 − 86.924 × T	[32]
	$\Phi_{\mathrm{Mg}}^{\mathrm{Al}}$ = −105,022.4 − 100.826 × T	[32]
	$\Phi_{\mathrm{Mg}}^{\mathrm{Zn}}$ = −97,239.0 − 87.338 × T	[32]
	$\Phi_{\mathrm{Mg}}^{\mathrm{Sn}}$ = −76,913.9 − 71.922 × T	[32]
	$\Phi_{\mathrm{Mg}}^{\mathrm{Mg},\mathrm{Al}}$ = 154,978.2	[32]
Mobility of Al	$\Phi_{\mathrm{Al}}^{\mathrm{Al}}$ = −115,705.9 − 104.143 × T	[32]
	$\Phi_{\mathrm{Al}}^{\mathrm{Mg}}$ = −133,378.9 − 86.232 × T	[32]
	$\Phi_{\mathrm{Al}}^{\mathrm{Zn}}$ = −97,239.0 − 87.338 × T	[32]
	$\Phi_{\mathrm{Al}}^{\mathrm{Sn}}$ = −76,913.9 − 71.922 × T	[32]
	$\Phi_{\mathrm{Al}}^{\mathrm{Mg},\mathrm{Al}}$ = 125,172.6	[32]
	$\Phi_{\mathrm{Al}}^{\mathrm{Mg},\mathrm{Zn}}$ = 313,977.051	This work
	$\Phi_{\mathrm{Al}}^{\mathrm{Mg},\mathrm{Sn}}$ = 214,599.609	This work
Mobility of Zn	$\Phi_{\mathrm{Zn}}^{\mathrm{Zn}}$ = −97,239.0 − 87.338 × T	[32]
	$\Phi_{\mathrm{Zn}}^{\mathrm{Mg}}$ = −125,731.0 − 76.734 × T	[32]
	$\Phi_{\mathrm{Zn}}^{\mathrm{Al}}$ = −115,705.9 − 104.143 × T	[32]
	$\Phi_{\mathrm{Zn}}^{\mathrm{Sn}}$ = −76,913.9 − 71.922 × T	[32]
	$\Phi_{\mathrm{Zn}}^{\mathrm{Mg},\mathrm{Zn}}$ = 80,988.7	[32]
	$\Phi_{\mathrm{Zn}}^{\mathrm{Mg},\mathrm{Al}}$ = 90,957.031	This work
	$\Phi_{\mathrm{Zn}}^{\mathrm{Mg},\mathrm{Sn}}$ = −11,209.270	This work
Mobility of Sn	$\Phi_{\mathrm{Sn}}^{\mathrm{Sn}}$ = −76,913.9 − 71.922 × T	[32]
	$\Phi_{\mathrm{Sn}}^{\mathrm{Mg}}$ = −143,787.3 − 72.615 × T	[32]
	$\Phi_{\mathrm{Sn}}^{\mathrm{Al}}$ = −115,705.9 − 104.143 × T	[32]
	$\Phi_{\mathrm{Sn}}^{\mathrm{Zn}}$ = −97,239.0 − 87.338 × T	[32]
	$\Phi_{\mathrm{Sn}}^{\mathrm{Mg},\mathrm{Sn}}$ = −162,023.5	[32]
	$\Phi_{\mathrm{Sn}}^{\mathrm{Mg},\mathrm{Al}}$ = 191,345.215	This work

## Data Availability

The data presented in this study are available upon reasonable request from the corresponding author.

## Supplementary Materials

The following are available online at https://www.mdpi.com/article/10.3390/ma15010283/s1, Figure S1: Model-predicted composition–distance profiles of different hcp Mg–Al–Zn diffusion couples annealed at (a) 663 K for 144 h and (b) 708 K for 144 h as well as an enlarged composition–distance curve of Zn annealed at (c) 663 K for 144 h and (d) 708 K for 144 h from the present atomic mobilities (solid lines) compared with those of Zhong et al. [32] (dashed lines) and the experimental data [33] (symbols).
