# Supplementary material for: An Effective Strategy to Maintain the CALPHAD Atomic Mobility Database of Multicomponent Systems and Its Application to Hcp Mg–Al–Zn–Sn Alloys
Source: Materials (Basel). 2021 Dec 31;15(1):283. doi: 10.3390/ma15010283 (PMC8746026; doi:10.3390/ma15010283)
Supplement: Supplementary file 1 [file materials-15-00283-s001.zip › materials-1455510-supplementary.pdf]

materials

MDPI

*Supplementary Materials*

# An Effective Strategy to Maintain the CALPHAD Atomic Mobility Database of Multicomponent Systems and Its Application to Hcp Mg–Al–Zn–Sn Alloys

**Ting Cheng, Jing Zhong and Lijun Zhang ***

State Key Laboratory of Powder Metallurgy, Central South University, Changsha 410083, China; chengting@csu.edu.cn (T.C.); zhongjingjogy@csu.edu.cn (J.Z.)
* Correspondence: lijun.zhang@csu.edu.cn

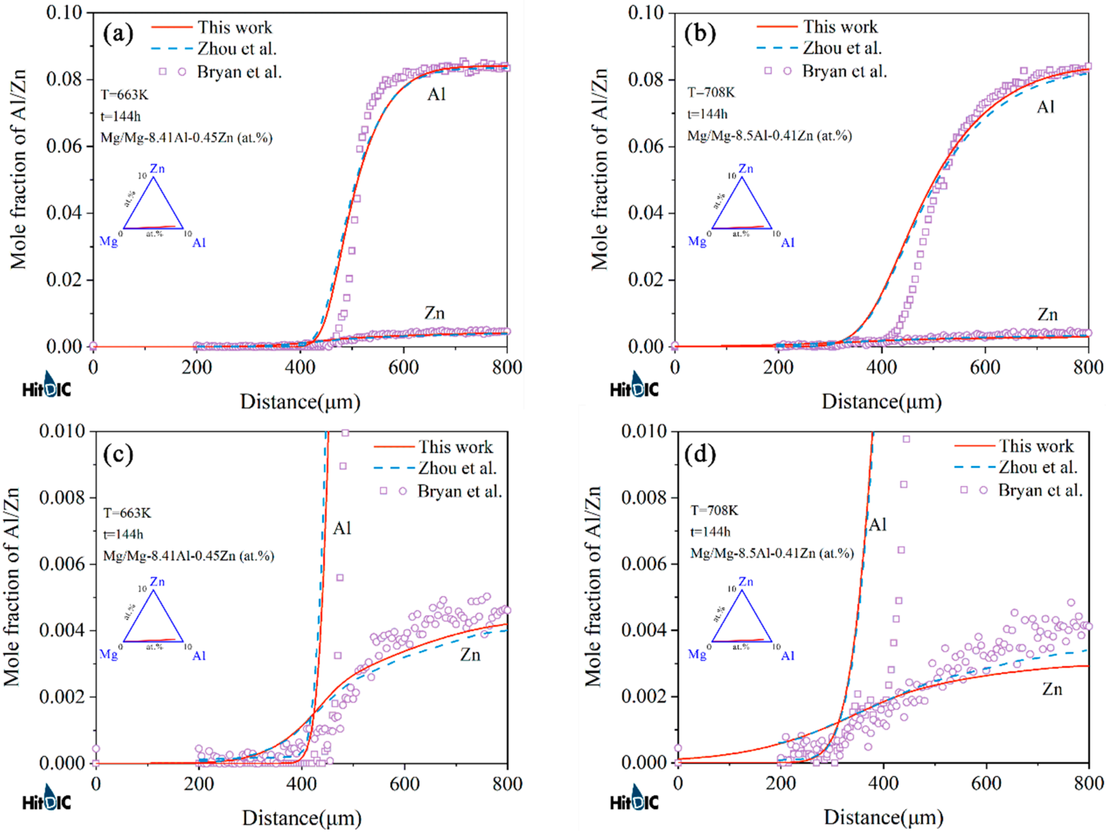


**Figure S1.** Model-predicted composition-distance profiles of different hcp Mg-Al-Zn diffusion couples annealed at (**a**) 663 K for 144 h, and (**b**) 708 K for 144 h, as well as enlarged composition-distance curve of Zn annealed at (**c**) 663 K for 144 h, and (**d**) 708 K for 144 h, due to the present atomic mobilities (solid lines), compared with those of Zhong et al. [32] (dashed lines) and the experimental data [33] (symbols).
